# Supplementary material for: Chitosan Oligosaccharide Ameliorates Nonalcoholic Fatty Liver Disease (NAFLD) in Diet-Induced Obese Mice
Source: Mar Drugs. 2019 Jul 2;17(7):391. doi: 10.3390/md17070391 (PMC6669476; doi:10.3390/md17070391)
Supplement: Supplementary file 1 [file marinedrugs-17-00391-s001.zip › Supplementary materials/Supplementary Materials Table S1.docx]

Supplementary Materials Table S1. The constitutions of high-fat diet

| Ingredient | Content (g) |
| --- | --- |
| Casein, 80 Mesh | 200 |
| L-Cystine | 3 |
| Corn Starch | 0 |
| Maltodextrin 10 | 125 |
| Sucrose 68.8 | 68.8 |
| Cellulose, BW200 | 50 |
| Soybean Oil | 25 |
| Lard | 245 |
| Mineral Mix S10026 | 10 |
| DiCalcium Phosphate | 13 |
| Calcium Carbonate | 5.5 |
| Potassium Citrate.1H_2_O | 16.5 |
| Vitamin Min V10001 | 10 |
| Choline Bitartrate | 2 |
| FD&C Blue Dye #1 | 0.05 |
| Total | 773.85 |
