# Supplementary figures and images for: Chitosan Oligosaccharide Ameliorates Nonalcoholic Fatty Liver Disease (NAFLD) in Diet-Induced Obese Mice
Source: Mar Drugs. 2019 Jul 2;17(7):391. doi: 10.3390/md17070391 (PMC6669476; doi:10.3390/md17070391)

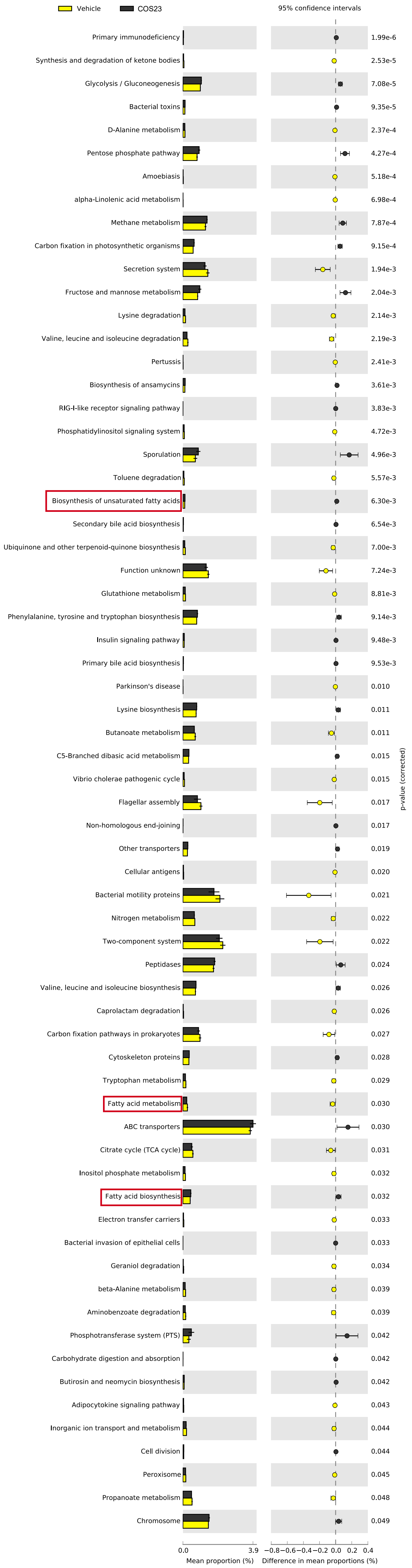

Supplement: Supplementary file 1 [file marinedrugs-17-00391-s001.zip › Supplementary materials/Supplementary Materials Figure S1.pdf]
